# Reversion of Val(Ganciclovir)-Resistance-Associated Mutations in Two SOT Patients with Mismatched Serostatus for CMV (D+/R-)

**DOI:** 10.3390/v17111462

**Published:** 2025-10-31

**Authors:** Elena Seminari, Alessandra Tebaldi, Aurelia Sangani, Paola Giordani, Daniele Lilleri, Stefania Paolucci, Giulia Campanini, Elizabeth Iskandar, Fausto Baldanti, Raffaele Bruno

**Affiliations:** 1 Infectious Diseases Unit, Fondazione IRCCS Policlinico San Matteo, 27100 Pavia, Italy; aurelia.sangani01@universitadipavia.it (A.S.); paola.giordani01@universitadipavia.it (P.G.); r.bruno@smatteo.pv.it (R.B.); 2 Infectious Diseases Unit, ASST Papa Giovanni XXIII, 24100 Bergamo, Italy; atebaldi@asst-pg23.it; 3 Microbiology and Virology Unit, Fondazione IRCCS Policlinico San Matteo, 27100 Pavia, Italy; d.lilleri@smatteo.pv.it (D.L.); s.paolucci@smatteo.pv.it (S.P.); g.campanini@smatteo.pv.it (G.C.); e.iskandar@smatteo.pv.it (E.I.); f.baldanti@smatteo.pv.it (F.B.); 4 National PhD Programme in One Health Approaches to Infectious Diseases and Life Science Research, Department of Public Health, Experimental and Forensic Medicine, University of Pavia, 27100 Pavia, Italy; 5 Department of Clinical, Surgical, Diagnostic and Pediatric Sciences, University of Pavia, 27100 Pavia, Italy

**Keywords:** cytomegalovirus, transplantation, antiviral resistance, maribavir, letermovir

## Abstract

The emergence of drug-resistant cytomegalovirus (CMV) complicates viral response to therapy. We present two cases of solid organ transplant (SOT) recipients, highlighting the reversion of UL97 mutations associated with val(ganciclovir) resistance. Patient 1, a heart transplant recipient, initially received pre-emptive treatment with val(ganciclovir), followed by foscarnet for recurrent CMV episodes. Mutations A594V in the UL97-kinase gene and V715M in the UL54-polymerase gene were detected. He developed CMV colitis and was then treated with maribavir. After discontinuing val(ganciclovir), genotyping revealed no resistance mutations. Following CMV DNA suppression, secondary prophylaxis with letermovir and val(ganciclovir) was initiated. Patient 2, a double-lung transplant recipient, experienced several CMV episodes. Initially treated with val(ganciclovir), he developed the L595S mutation in the UL97 kinase gene, conferring resistance. Therapy was then switched to foscarnet, which was suspended due to renal failure, and then to maribavir. Subsequently, the H411Y mutation in the UL97 was detected, conferring maribavir resistance, while val(ganciclovir) mutation was no longer detectable. He was then treated with val(ganciclovir) and letermovir, achieving undetectable CMV DNA, and then continued letermovir alone as prophylaxis. Detecting gene mutations that confer drug resistance is crucial for managing antiviral therapy when virological response is lacking. In our cases, the reversion of (va)ganciclovir-resistance mutations occurred after drug withdrawal, a previously unreported finding.

## 1. Introduction

Cytomegalovirus (CMV) infection remains a leading cause of morbidity and mortality after solid organ transplantation (SOT), particularly in CMV seronegative recipients of organs from CMV seropositive donors (CMV D+R-) [1]. The mainstay of first-line therapy for CMV disease is (val)ganciclovir. [2]. A lack of virological response after 2–3 weeks of (val)ganciclovir treatment is defined as a resistant/refractory infection, which could be attributed to the emergence of drug-resistant CMV strains or to other causes such as poor bioavailability or inadequate dosing [3,4]. Mutations conferring resistance to (val)ganciclovir develop in up to 12% of CMV D+R- patients [5]. Second-line therapies for drug-resistant CMV infection include foscarnet and cidofovir, but their use is limited by the need for intravenous administration and the side effects, including nephrotoxicity and neuropathy [6]. Occasionally, resistance to either foscarnet or cidofovir has also been observed due to mutations in the UL54 gene [7]. Recently, maribavir and letermvoir, drugs with novel antiviral targets, have been introduced in the treating algorithm for high-risk SOT patients [5,6,7,8]. Letermovir exhibits a low viral genetic barrier for the development of resistance in clinical practice when used for treatment of actively replicating CMV infection rather than in the prophylaxis of CMV infection, and cases of resistance to maribavir have also been observed [9]. Few data exist in the literature regarding the most effective strategy to be adopted in the treatment of drug-resistant CMV infections occurring in high-risk patients (CMV D+/R-) [9]. To our knowledge, reversion of mutations conferring resistance to first-line drugs has not yet been reported.

We present two case reports of high-risk SOT recipients (CMV D+/R-) with CMV strains harboring UL97 mutations conferring resistance to val(ganciclovir)and reverted during follow-up, thereby enabling the re-use of val(ganciclovir) as rescue therapy.

## 2. Case Reports

Case 1: A 30-year-old male with Danon disease received heart transplant in October 2021 (CMV serostatus D+/R-). Due to the occurrence of multiple episodes of CMV infection in 2022, he was pre-emptively treated with (val)ganciclovir at therapeutic dose and subsequently with foscarnet. As mutations associated with resistance to both drugs were sequentially detected (A594V mutation in the UL97-kinase gene and V715M mutation in the UL54-polimerase gene) in April 2022, in the absence of T cell-mediated immunity, he was finally treated with maribavir in August 2022. Blood CMV DNA was 123,750 copies/mL, and he was suffering from CMV-associated colitis (CMV cell-mediated immunity remained undetectable). Furthermore, since (val)ganciclovir suspension, (val)ganciclovir resistance-associated mutations were no longer detected in genotyping tests performed since July 2022. After 8 weeks of treatment with maribavir, blood CMV DNA was 2700 copies/mL. Maribavir was then discontinued, and letermovir in combination with (val)ganciclovir was prescribed as secondary prophylaxis. (Val)ganciclovir was discontinued after eleven weeks, when CMV DNA levels stabilized at a low level (1560 copies/mL). In May 2023, blood CMV DNA was 1615 copies/mL, while the same sample pretreated with DNAse tested negative. Letermovir was discontinued after nineteen months of treatment, and his plasma CMV DNA has remained persistently undetectable in the 12-month follow-up. Since August 2022, a gradual recovery of anti-CMV cell-mediated immunity had been observed during prophylaxis with letermovir, with recovery of CMV-specific immunity documented in February 2025.

Case 2: A 60-year-old male received double-lung transplantation for idiopathic pulmonary fibrosis in May 2021. He has been partially described elsewhere [10]. In the following months, he developed several CMV infection episodes treated on a pre-emptive basis with (val)ganciclovir. Seven months later, a mutation in UL97 conferring (val)ganciclovir resistance (L595S) was detected. Consequently, (val)ganciclovir was replaced by foscarnet, which was subsequently discontinued due to renal failure. In June 2023, treatment with maribavir was started when CMV DNA was 359,100 copies/mL. In the following months, gene mutations associated with (val)ganciclovir resistance were no longer detectable by genotyping tests. During treatment with maribavir, CMV DNAemia was persistently positive, remaining above 100,000 copies/mL. After 13 months of maribavir treatment, H411Y mutation in UL97 that confers resistance to maribavir was detected, while T-cell response to CMV remained undetectable and blood CMV DNA was 107,910 copies/mL. Immunosuppressive regimen was shifted from tacrolimus plus mycophenolate to everolimus. To ensure a reduction in blood CMV DNA, the patient was then treated with (val)ganciclovir at therapeutic dosage for twelve weeks. When CMV DNAemia reached 18,930 copies/mL, letermovir was added to the ongoing (val)ganciclovir therapy. After 2 months of combination treatment, the patient continued letermovir alone as secondary prophylaxis. Seven months later, he remained on letermovir, with persistently low levels of DNAemia and undetectable CMV RNA. In January 2025, CMV DNA viremia was 2330 copies/mL, while blood CMV RNA remained undetectable.

## 3. Discussion

CMV infection resistant to (val)ganciclovir is a rare condition, reported in less than 2% of SOT recipients, and mainly in the high-risk population (D+/R-) [5]. Cases of double resistance to valganciclovir and foscarnet, as observed in our patients, have been reported [11]. Treatment of CMV infection resistant to antiviral(s) in SOT recipient patients still represents a challenge for clinicians, as patients are at increased risk of graft failure, nephrotoxicity and prolonged hospitalization [12,13,14]. The major concern with the use of newer available drugs (i.e., maribavir and letermovir) in these patients is represented by the emergence of resistant variants.

Maribavir-associated mutations can arise in the context of high viral loads [15,16]. Moreover, resistance can develop during periods of intensified immunosuppression, such as during treatment for rejection [17].

Although the use of letermovir as secondary prophylaxis in heart and lung transplant recipients is still off-label, some case series and retrospective studies have been published [18,19]. Due to its low viral genetic barrier, letermovir is less effective for the treatment of active CMV infection, particularly in cases of high-level CMV DNAemia [20]. Moreover, given letermovir’s mechanism of action, monitoring CMV DNAemia by conventional PCR assays in patients receiving letermovir may not be indicative of viral replication. Therefore, in these cases, it is necessary to assess active replication by evaluating CMV DNA after digestion with DNAase or by quantifying viral RNA. Letermovir inhibits capsid-filling, but has little effect on DNA levels in cells undergoing lytic replication. Virion-encapsidated DNA remains detectable after DNAse treatment of plasma, while non-infectious free-floating DNA derived from infected cell lysis is degraded [21,22]. Similarly, detection in plasma of UL21.5 mRNA, which was found to be packaged into virions, indicates the presence of infectious virus particles [23,24].

In the salvage setting, combination or sequential administration of anti-CMV drugs has been proposed to reduce the risk of resistant strain selection [5,25,26]. In particular, the combination of letermovir with (val)ganciclovir or foscarnet has been employed in the management of R/R CMV infection in the transplant setting [5,27].

As with other viral infections, such as HIV and HCV, sequential genotyping tests to detect mutations at baseline and during therapy are of critical importance for managing high-risk SOT patients [15]. A reversion of (val)ganciclovir resistance-associated mutation has been described following discontinuation of (val)ganciclovir in a patient with primary immunodeficiency [25]. This could have resulted from reactivation of a (val)ganciclovir-sensitive virus residing in a latent reservoir.

In our two cases, sequential genotyping performed during the prolonged course of CMV infection showed reversion of mutations associated with (val)ganciclovir in both cases after a period of drug withdrawal. As treatment options became limited, we leveraged the genotyping data to recycle (val)ganciclovir. In the first case, (val)ganciclovir was combined with letermovir during the first weeks of treatment to reduce the risk of selecting resistance mutations to letermovir. In the second case, (val)ganciclovir was reintroduced as monotherapy after genotypic reversion, to reduce CMV DNA levels that allowed the safe use of letermovir as secondary prophylaxis.

Although we found no literature regarding genotypic reversion of CMV-associated mutations that confer resistance to (val)ganciclovir, we can hypothesize a mechanism similar to that observed in other viruses, such as human immunodeficiency virus (HIV). In that context, it is well established that, in the absence of drug pressure, the wild-type strain, which has a higher replicative capacity than mutated strains, may re-emerge [28]. However, it is important to note that in the case of HIV, previously acquired resistance mutations not detected by genotypic testing can be archived within the latent viral reservoirs and may reappear upon reintroduction of the implicated drug, leading to therapeutic failure [29]. We cannot exclude that this phenomenon may also occur in other viruses capable of establishing a latent infection, such as CMV. However, re-emergence of mutated strains was not observed in our patients. In the study by Bravo et al., various mutations in different targets of antiviral drugs were analyzed, showing their ability to confer low, moderate, or high levels of resistance and it was demonstrated that some of these mutations, such as G441S and A543V in UL54 and F345L and P800L in UL56, are associated with a reduced replicative capacity compared to the wild-type strain [7]. It is therefore crucial to study the phenomenon of resistance reversion in order to understand the best management strategy for refractory/resistant CMV infection, particularly in cases where maribavir is ineffective due to the emergence of specific resistant strains or its low penetration into the involved organs (e.g., retinitis, encephalitis). One strategy that requires validation is combination therapy; in fact, in vitro studies have shown an additive effect when combining ganciclovir with foscarnet or letermovir [30]. Based on these results, we chose to use the combination of ganciclovir and letermovir in our patients. The combination of both drugs has already been reported as effective in clinical practice in selected cases [26,31,32].

## 4. Conclusions

Detection of gene mutations that confer drug resistance is a cornerstone of antiviral therapy management when a lack of virological response is observed. In the two reported cases, letermovir proved effective in controlling CMV infection as secondary prophylaxis, and reversion of (val)ganciclovir-resistance-associated mutations was observed after (val)ganciclovir withdrawal. This novel observation has never been previously reported and can be considered in the management of antiviral treatment for CMV infections in D+/R- SOT.

## Data Availability

The data presented in this study are available on request from the corresponding author due to ethical reasons.

## Abbreviations

The following abbreviations are used in this manuscript:

CMV	cytomegalovirus
SOT	Solid organ transplantation
CMV D+R-	CMV seronegative recipients of organs from CMV seropositive donors
HIV	Human Immunodeficiency Virus
